# First episode of psychiatric and neuropsychiatric disease among patients infected with COVID‐19: A scoping review

**DOI:** 10.1002/pcn5.70146

**Published:** 2025-06-25

**Authors:** Wali Yousufzai, Alex Heo, Kyle Gu, Edward Sun, Gabriel Lopez, Shreya Balamurali, Jennifer Adjei‐Mosi, Riley Shin, Daniel B. Stuart, Peggy Edwards, Regina Baronia, Wail Amor, Terry McMahon

**Affiliations:** ^1^ Psychiatry Department Texas Tech University Health Sciences Lubbock Lubbock Texas USA; ^2^ Nassau University Medical Center East Meadow New York USA

**Keywords:** COVID‐19, depression, neurocognitive disorder, neuropsychiatry

## Abstract

This scoping review aims to examine the frequency and prevalence of neuropsychiatric disorders reported in patients infected with coronavirus disease 2019, and the mechanisms by which these develop during and post infection. A systematic search using relevant search terms and key words was done on six electronic databases of literature on neuropsychiatric conditions post‐coronavirus disease 2019 infection from 2020 to 2023. Data were extracted following Joanna Briggs Institute guidelines, focusing on key findings, intervention details, and outcomes. We included 333 studies in the review. Studies indicated an elevated risk of neuropsychiatric disorders post‐coronavirus disease 2019, with some risks remaining high 2 years after diagnosis. A significant prevalence of depressive, psychotic, and anxiety disorders, as well as post‐traumatic stress symptoms were noted among coronavirus disease 2019 survivors. There was increased prevalence of insomnia and other sleep disturbances, mild to severe cognitive dysfunction, and eating disorders. Coronavirus disease 2019 infection is associated with a significant risk of developing various neuropsychiatric disorders, including schizophrenia, depressive disorders, anxiety, post‐traumatic stress disorder, and cognitive dysfunction. Long‐term monitoring and early interventions are essential to mitigate these risks and improve patient outcomes.

## INTRODUCTION

The objectives of this review were to map the breadth of neuropsychiatric sequelae associated with coronavirus disease 2019 (COVID‐19) infection, identify the range of symptoms and disorders reported, understand the underlying mechanisms, and highlight gaps in the current literature.

The ramifications of the COVID‐19 pandemic caused by the SARS‐CoV‐2 virus have affected populations and individuals in various spheres, with a significant impairment in global mental and physical health. Studies have shown the prevalence of neuropsychiatric symptoms in patients diagnosed with acute COVID‐19 infection. These include anxiety, depression, nightmares, suicidality, insomnia, and musculoskeletal disorders. Prior to the pandemic, it was well‐established that viral infections could lead to neuropsychiatric manifestations, often through mechanisms such as direct viral invasion of the central nervous system (CNS), immune‐mediated responses, and systemic inflammation. However, the specific and widespread impact of COVID‐19 infection on mental health and neurological function has generated unprecedented concern and necessitated a comprehensive examination.

While data have focused on the effects of acute COVID‐19 on neuropsychiatric disorders, we are now experiencing the later stages of the pandemic where we are also able to observe long‐term effects of the disease. The effects of COVID‐19 infection 4 weeks post infection and/or hospitalization are termed “long COVID,” indicating the persistence of symptoms 3 weeks to several months later.^1^ Neuropsychiatric symptoms present in patients with long COVID can be similar to or differ from those present during the initial SARS‐CoV‐2 infection period, including psychiatric disorders such as post‐traumatic stress disorder (PTSD), anxiety, depression, insomnia, suicidality, and amnesia.^1^

The prevalence of neuropsychiatric symptoms could be caused by the neuro‐invasive and neurotropic properties of COVID‐19 infection, indicating its long‐term impact on mental health.^2^, ^3^ Specifically, research has identified PTSD as a prevalent post‐infection symptom in long COVID patients, with 30.2% of post‐COVID‐19 patients affected by the disorder.^4^ Due to the possibility of worsening symptoms, hospitalization, and death in patients with long‐COVID‐related neuropsychiatric illness, it is important to study the relationship between the two and identify possible interventions and treatments for such patient populations.

The aim of this scoping review was to address the broad and diverse neuropsychiatric consequences of COVID‐19 infection because it maps the existing literature and identifies the extent and nature of research activity in this emerging field. It is particularly useful for identifying significant gaps in the literature, given the novelty of COVID‐19 infection and its long‐term neuropsychiatric effects, thereby guiding future research priorities and funding. The complex and multifactorial mechanisms underlying the impact of COVID‐19 on the CNS and mental health can be better understood by synthesizing diverse study designs and methodologies through a scoping review.

Additionally, the heterogeneity of existing studies, which vary in population, settings, and outcomes, can be accommodated to provide a holistic understanding of the evidence landscape. Ultimately, collating and summarizing the current state of knowledge through this scoping review lays the foundation for more detailed systematic reviews and meta‐analyses, addressing specific questions with greater precision.

## METHODS

*Protocol and registration:* The protocol is registered in the Open Science Framework at osf.io under the project name “A Scoping Review‐First Episode of Psychiatric and Neuropsychiatric Disease Among Patients Infected with Covid‐19”, registration https://doi.org/10.17605/OSF.IO/5VJN2.

### Eligibility criteria

Data from all studies from the beginning of the pandemic in January 2020 onward that include suspected and/or confirmed COVID‐19 cases with neuropsychiatric and psychiatric presentations from all age groups were collected. Exclusion criteria: non‐human studies, studies before January 2020, reviews and meta‐analyses that do not include direct human subject exposure, full article not available (e.g., poster presentations, conference abstracts), relevant narrative reviews, opinion papers, editorial and clinical guidelines.

### Search strategy and information sources

Electronic database searches were conducted by the reference librarian/medical informationist at the Preston Smith Library of the Health Sciences at Texas Tech University Health Sciences Center in Lubbock, Texas. The baseline search strategy was developed in PubMed and Ovid Medline, and was conducted in consultation with the investigators. The OVID Medline, Cochrane Central Register of Controlled Trials, and PsycInfo searches were conducted through the OVID platform on September 16, 2022. Embase® was searched through the Elsevier platform. LitCovid was searched through the National Institute of Health National Library of Medicine platform on December 22, 2022. All results were downloaded into Covidence systematic review software, with automatic deduplication, for screening conducted by the investigators team. The complete search strategies for all databases are available through Open Science Framework at osf.io.

### Study screening and selection

The Covidence systematic review software was utilized for the importation, screening, filtration, and data extraction from all pertinent studies with the assistance of a medical reference librarian. From the sources mentioned above, 8752 studies were discovered. Following this, full‐text articles were attached to potentially eligible studies within Covidence. Two authors independently assessed these based on official extraction criteria and a third author conducted the conflict resolution. Any discrepancies among reviewers were addressed through discussion and, if necessary, with the help of a third senior researcher. Ultimately, 333 studies were incorporated into the review (Table 1).

**Table 1:** The PRISMA flow diagram outlines the search results for this scoping review.

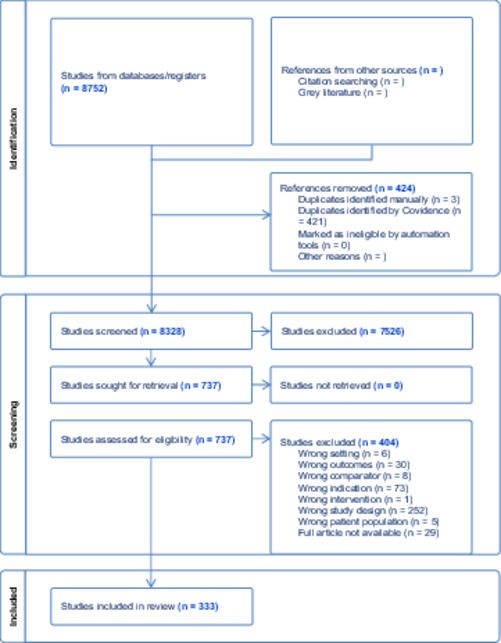

TABLE 1 A total of 8752 articles were sourced from multiple databases: OVID Medline, Cochrane Central Register of Controlled Trials, Embrase, LitCovid, and PsycInfo. A total of 8328 articles were left for screening after deduplication. The titles and articles were then screened by two independent reviewers, ensuring the highest level of objectivity for assessment against the inclusion criteria for the review. Full‐text versions of 737 articles were compiled, of which a significant 333 articles were included in this scoping review, each contributing valuable insights to the field.

### Data extraction and analysis

Data were extracted from articles included in the scoping review using the Covidence software data extraction self‐guided template. We followed the Joanne Briggs Institute (JBI) identifiers explained in JBI's chapter 11 scoping review guideline. The following data were extracted according to the JBI scoping review guideline: author(s), identification features of report, year of publication, aims/purpose, population and sample size within the source of evidence, methodology, intervention type, comparator and details of intervention (i.e., duration of the intervention), outcomes and details of these (i.e., how measured), and key findings that related to the scoping review question/s. Lastly, we extracted information regarding the study outcomes.

## RESULTS

From an initial pool of 8752 studies, 8328 were screened, 7526 were excluded, and 737 full texts were reviewed, resulting in 333 studies being included in the review. The results indicated an elevated risk of neuropsychiatric disorders post‐COVID‐19 infection. Depressive disorders were significantly prevalent among COVID‐19 survivors, with neuroimaging studies correlating depressive symptoms to changes in brain regions. Although anxiety prevalence was high post intensive care unit (ICU) treatment, it decreased over time. Post‐traumatic stress symptoms were notably high among survivors, especially those with severe symptoms or pre‐existing mental health conditions. Insomnia and other sleep disturbances were prevalent, significantly impacting quality of life. Cognitive dysfunction, ranging from mild to severe, positively correlated with the severity of the initial infection. Additionally, emerging eating disorders, including anorexia nervosa, were observed in children and adolescents post‐COVID‐19 infection.

The studies used for this scoping review were conducted in various locations around the world. Studies from beginning of pandemic in January 2020 onward that include suspected and/or confirmed COVID‐19 cases with neuropsychiatric and psychiatric presentations were collected. Study characteristic are illustrated in Table 1. Among the 55 different countries that participated to the results of this review, we list only those with the greatest number of studies: Italy (61), followed by United States (48), China (24), Germany (21), Spain (15), and Brazil (15). All the studies included males and females from variable age groups, encompassing diverse races and ethnicities.

**Figure 1:**
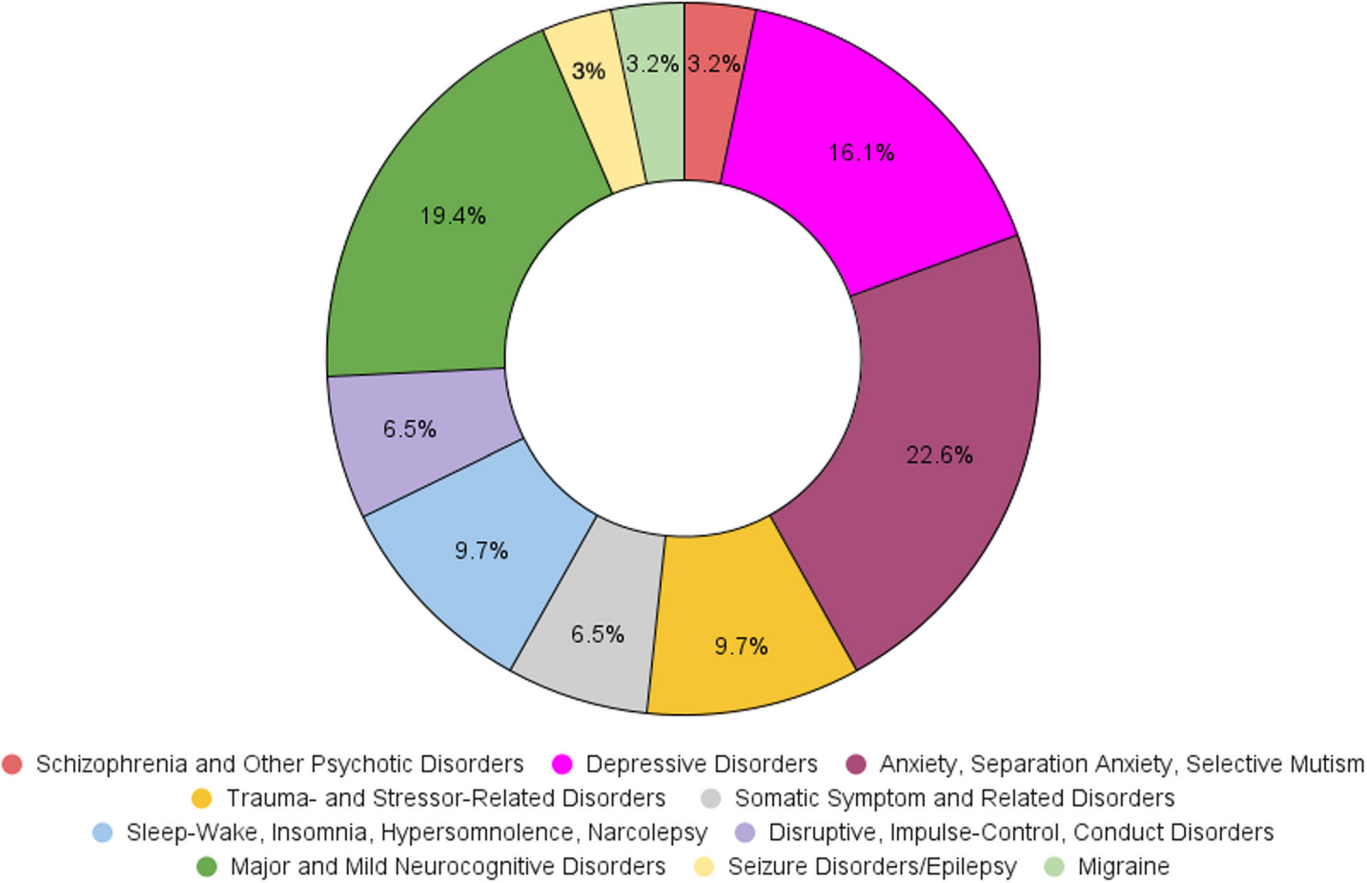
The distribution of neuropsychiatric outcomes among COVID‐19 patients in 2020. Anxiety disorder and separation anxiety were the most frequently reported conditions, followed by major and mild neurocognitive disorders and major depressive disorders.

**Figure 2:**
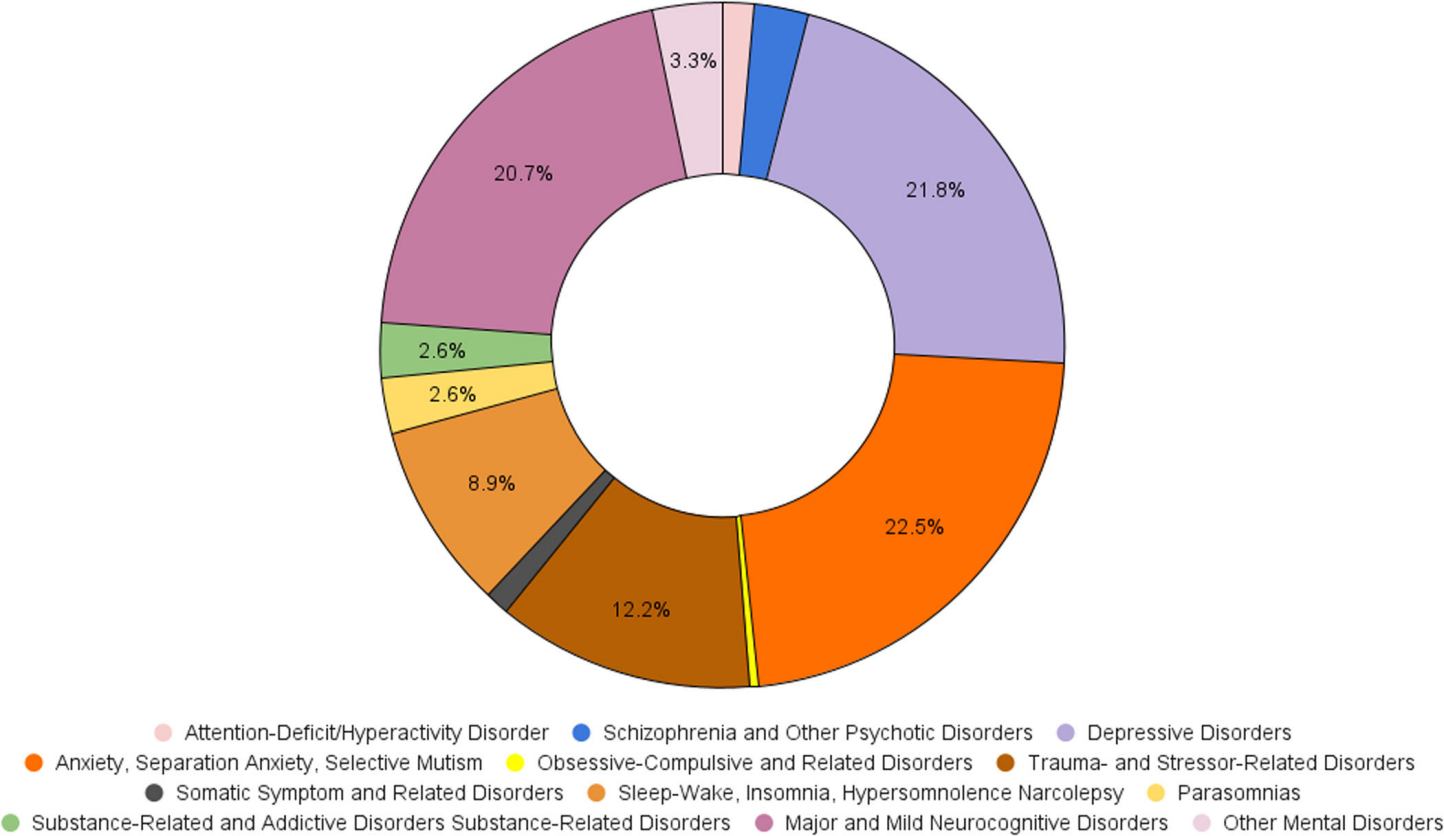
The neuropsychiatric sequelae reported in 2021. Anxiety disorder and separation anxiety remained the most prevalent, with depressive disorders as the second most common outcome, and major and mild neurocognitive disorders as the third.

**Figure 3:**
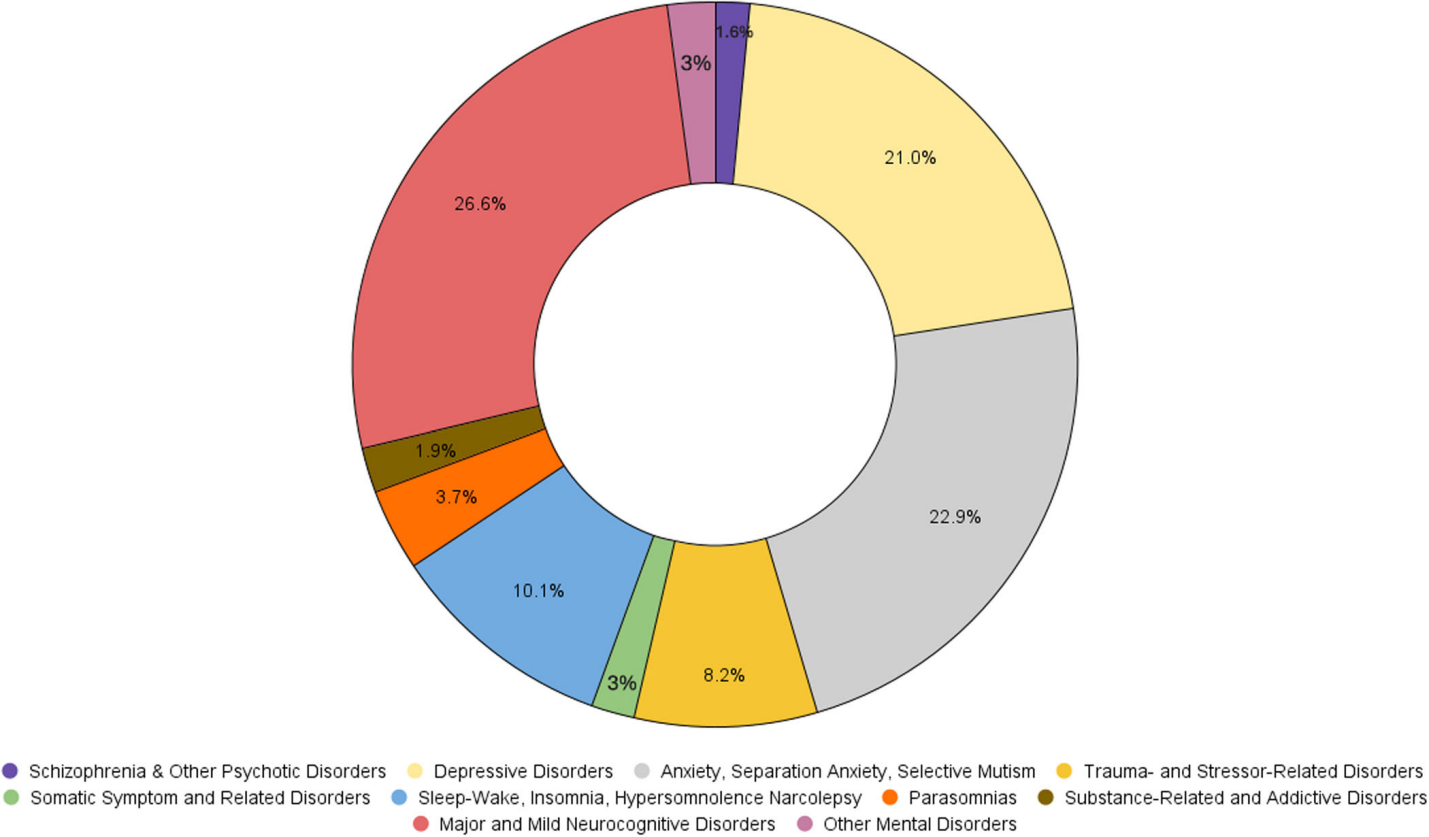
The distribution of neuropsychiatric sequelae in 2022, where major and mild neurocognitive disorders were reported at the highest rates, surpassing other conditions.

**Figure 4:**
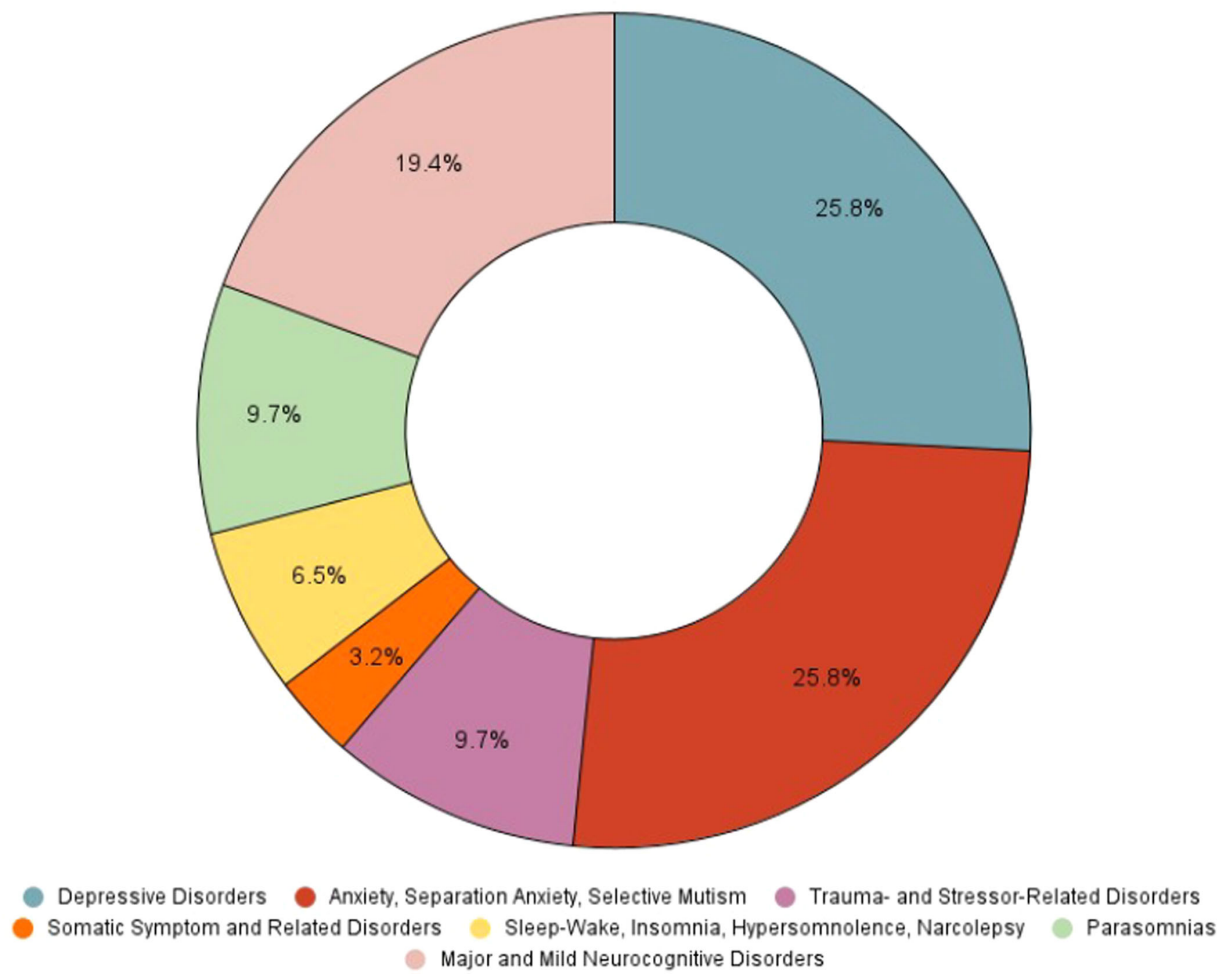
In 2023 anxiety disorder, separation anxiety, and depressive disorders were the most prevalent neuropsychiatric outcomes among COVID‐19 patients, indicating a shift in the pattern of reported conditions over the years.

Figures 1, 2, 3, 4 illustrate the progression and prevalence of neuropsychiatric sequelae following COVID‐19 infection from 2020 to 2023. The data highlight the changing landscape of mental health outcomes over this period. In 2020, anxiety disorder emerged as the most frequently reported neuropsychiatric condition, closely followed by major and mild neurocognitive disorders as the second most common, and major depressive disorders ranking third (Figure 1). As the pandemic progressed into 2021, anxiety disorder continued to be the predominant sequela, with depressive disorders becoming the second most prevalent, and major and mild neurocognitive disorders shifting to the third position (Figure 2). By 2022, there was a notable shift, with major and mild neurocognitive disorders being reported at the highest rates among neuropsychiatric outcomes, surpassing anxiety‐related conditions during this year (Figure 3). However, by 2023, anxiety disorder, separation anxiety, and depressive disorders became the leading neuropsychiatric outcomes, indicating a resurgence and persistence of anxiety and mood‐related disorders as major sequelae of COVID‐19 infection (Figure 4). This evolving pattern underscores the dynamic impact of the pandemic on mental health, necessitating ongoing monitoring and adaptation of mental health resources and interventions (Table 2).

**Table 2:** Study characteristics.

Characteristic	*n*
*Year of publication*	
2020	15
2021	104
2022	202
2023	12
*Age range (number of studies may overlap)*	
>18 years	8
18–64 years	317
*Study design*	
Randomized controlled trial	3
Cohort study	218
Case control study	31
Case series study	5
Cross sectional study	72
Other: exploratory	2
Other: retrospective review	2
*Countries contributed to results of review*	55

### Schizophrenia spectrum and other psychotic disorders

The review of 15 studies on schizophrenia spectrum and other psychotic disorders in the context of COVID‐19 highlights significant findings. One study reported a large effect size in the Schizophrenia Thought Disorder subscale (SCZ‐T), suggesting that sociocultural, environmental, and psychosocial stressors may contribute to post‐COVID‐19 psychiatric sequelae.^5^ Additionally, the risk for developing a psychotic disorder remains elevated for up to 2 years post COVID‐19 diagnosis.^6^, ^7^ Studies also indicated a nearly threefold increase in the likelihood of developing mental illnesses among COVID‐19 survivors, particularly for depression and psychosis, and increased the risk of hospitalization due to COVID‐19 in attention‐deficit/hyperactivity disorder (ADHD).^8^, ^9^, ^10^ The potential mechanisms for COVID‐19‐related psychiatric disorders include direct viral invasion of the central nervous system, immune overreaction, and increased pro‐inflammatory factors with higher rates of psychiatric hospitalizations.^11^, ^12^, ^13^, ^14^

### Depressive and anxiety disorders

A total of 192 out of 333 articles (observational studies; cross‐sectional, case control, retrospective cohort, prospective cohorts) reported on depressive disorders in confirmed cases of COVID‐19 infection and long COVID. Neuroimaging studies have found correlations between depressive scores and changes in brain regions such as the right hippocampus.^15^ Various studies reported the prevalence of major depressive disorder in patients with COVID‐19, with factors like restlessness, aggression, and severe mixed depression being common in these patients. Studies also showed a significant association between more severe depressive symptoms and post‐traumatic stress syndrome in COVID‐19 survivors with delayed recovery.^16^, ^17^, ^18^ Pregnant women with COVID‐19 had a higher risk of postpartum depression, and long COVID increased the risk of depression and anxiety.^19^, ^20^ Depression, anxiety, and altered mental status were more common and developed more quickly in patients with post‐acute‐COVID‐19 infection compared to similar symptoms in flue patients.^21^ Some studies found a correlation between biomarkers of inflammation and depressive symptoms, cognitive impairment, and other neuropsychiatric sequela in patients with COVID‐19.^22^, ^23^, ^24^, ^25^, ^26^, ^27^

#### Neuroimaging

The brainstem raphe (BR) alterations in transcranial sonography (TCS) of 70 patients were examined in a cross‐sectional study. Among these, 28.6% (*n* = 20) of long COVID‐19 patients exhibited reduced echogenicity of BR in the TCS examination. These patients had higher subscores for anxiety and depression (Hospital Anxiety and Depression Scale [HADS] depression: median 8 vs. 5.5, P = 0.006; HADS anxiety: median 9 vs. 6.5, P = 0.006) compared to normoechogenic patients. After adjusting for confounders, the odds ratio for relevant depressive symptoms was higher among long COVID patients with hypoechogenic raphe.^28^

#### Neuropsychiatric sequela of long COVID

The National Institute of Health defines long COVID, also known as post‐acute sequelae of SARS‐CoV‐2 infection, as a variety of symptoms that persist for weeks or months following the resolution of the acute phase of COVID‐19 infection. Out of 333 articles reviewed, 73 were identified that specifically investigated the sequelae of long COVID. In some studies, restlessness, aggregation, and high suicide risk, correlated with physical complaints and severe depression, were reported as long COVID sequalae.^29^, ^30^

#### Biomarkers correlated to COVID‐19 neuropsychiatric sequela

The systemic immune‐inflammation index, a measure of immune response and systemic inflammation based on peripheral lymphocyte, neutrophil, and platelet counts, was found to predict depressive symptoms, cognitive impairment processing speed, verbal memory and fluency, and psychomotor coordination predicted by baseline.^31^ Interleukin‐6 and C‐reactive protein were positively correlated with acute COVID‐19 symptoms, the number of medical comorbidities, and fatigue, and inversely correlated with measures of executive function.^32^ Multiplex cytokine and ultra‐sensitive interferon‐a2 measurements were similar between COVID‐19 long‐haulers and convalescent COVID‐19 individuals without persistent symptoms.^33^

Generalized anxiety disorder was one of the most frequently assessed and referenced neuropsychiatric disorders in this scoping review. An increased number of reported COVID‐related symptoms were associated with clinically significant levels of anxiety symptoms among depression and PTSD.^34^ In 2022, an Indian cohort study was launched to evaluate the mental health sequelae among ICU patients. The study found a high prevalence of anxiety and depression at discharge, with a significant drop in symptoms 2 months post discharge and a non‐significant decrease 6 months post discharge. These findings underscore the need for mental health strategies to support patient well‐being after ICU discharge.^35^ Mood disorders, anxiety and stress‐related disorders, and affective psychotic disorders were more prevalent among COVID‐19 survivors, whereas the increase in personality disorders was not statistically significant.^36^, ^37^

### Post‐traumatic stress disorder

A 2021 cross‐sectional study conducted in China investigated the prevalence of post‐traumatic stress symptoms and their impact on quality of life among COVID‐19 survivors. The researchers found a significantly higher prevalence of post‐traumatic stress symptoms among COVID‐19 survivors compared to the control group.^38^

In Norway, a 2021 study assessed the risk factors and prevalence of PTSD among hospitalized and non‐hospitalized COVID‐19 patients in 583 patients. The researchers determined that being female, being born outside Norway, and experiencing dyspnea were risk factors for persistent PTSD symptoms in both hospitalized and non‐hospitalized patients. Prior depression and the severity of COVID‐19 symptoms were also associated with PTSD in non‐hospitalized patients.^39^

### Somatic symptoms and migraines

Headaches, sensitivity disorders, and attention problems were common among persistent COVID‐19 symptoms. Sensory and motor complaints were more often linked to neurological diagnoses rather than post‐COVID‐19 syndrome, with elevated somatization levels suggesting a psychosomatic pathogenesis.^40^, ^41^, ^42^

Three articles (observational studies and cohort studies) reported on the relationship between migraines and COVID‐19 infection. Studies reported elevated headache frequency and worse quality of life related to their headaches, suggesting that COVID‐19 infection may impact headaches.^43^ There was a statistically significant increase in the number of headaches post COVID‐19 infection in patients with a prior history of migraines (mean 39.0 headaches/3‐month period) compared to those with no prior history of migraine headaches (mean 18.5 headaches/3‐month period).^43^

### Eating and sleep disorders

In a cohort study, researchers examined the emergence of eating disorders in children and adolescents who reported a loss of smell or taste after contracting COVID‐19.^44^ Out of the 84 patients in the study, 24 reported a loss of smell or taste following a confirmed COVID‐19 infection. Among these 24 patients, six developed a heightened focus on their eating habits, eventually culminating in anorexia nervosa. Conversely, in a cohort of 260,883 individuals with 6148 COVID‐19 survivors, findings indicated that COVID‐19 survivors were more prone to developing eating disorders among other psychiatric disorders.^45^

Insomnia and sleep disturbances are common neuropsychiatric sequelae of COVID‐19, with prevalence increasing significantly post COVID‐19 infection. In a cohort of 47 patients, insomnia prevalence rose from 10.6% to 27.3%, while 41.5% reported poor sleep quality.^46^ In a South Korean study of 6934 COVID‐19 survivors, 5.4% were diagnosed with insomnia disorder at a 6‐month follow‐up.^47^ Children previously hospitalized with COVID‐19 reported persistent fatigue, sleep disturbances, and sensory issues.^48^ High scores on insomnia severity and sleep quality indices during recovery indicate significant impacts on quality of life, with symptom duration correlating positively with insomnia development.^49^, ^50^, ^51^

### Delirium, neurocognitive disorders, seizure disorders, and Parkinson's disease

A total of 18 out of 333 articles (observational studies, cross‐sectional, retrospective, and prospective cohort studies, and one case series) reported on the presence of delirium and substance‐related and addictive disorders in patients with confirmed COVID‐19 infection. Psychological distress, lack of impulse control, and impaired emotional regulation and disinhibition were reported in some studies.^52^, ^53^ Multiple studies found a high prevalence of delirium in their samples, with those studies that involved elderly patients and those requiring ICU‐level hospitalization strongly correlated to the presence of delirium in association with COVID‐19 infection.^54^, ^55^, ^56^ One study found data to indicate a higher risk of developing substance‐related and addictive disorders following COVID‐19 infection. In a UK Biobank‐based cohort study of 26,181 COVID‐19 survivors, data showed that compared to controls, COVID‐19 survivors were at a significantly increased risk of substance use disorders, alcohol and tobacco use disorders.^57^

A total of 202 out of 333 articles (observational studies, cross‐sectional, case‐control, retrospective cohort, and prospective cohort) reported on the presence of major and mild neurocognitive disorders and sequelae in patients with confirmed COVID‐19 infection. As more than 60% of studies cited major and mild neurocognitive disorders following COVID‐19 infections, what is sometimes colloquially referred to as “long COVID” is a highly prevalent and an often‐experienced sequalae of COVID‐19 infection. Studies cite cognitive dysfunction in the acute phase up to more than 1 year following recovery from COVID‐19 infection.^43^, ^57^, ^58^, ^59^ Routine tests like the Montreal Cognitive Assessment, digit symbol, and phonetic verbal fluency test may be used to identify and distinguish patients who are experiencing significant post‐COVID‐19 cognitive impairment.^60^, ^61^, ^62^, ^63^ Altered consciousness and its progression had a direct link with death in COVID‐19 without focal neurological deficits in neuroimaging.^64^, ^65^, ^66^

Seven studies (observational studies, cohort studies, and one case series) analyzed the influence of COVID‐19 infection on seizure disorders. In a cohort study of 1,284,437 patients, risk of epilepsy or seizures remained increased at 2 years after a COVID‐19 diagnosis compared to the risk of other diagnoses (notably, mood and anxiety disorders), which subsided early.^67^

While only seven of 333 articles discussed the relationship between COVID‐19 infection and seizure disorders, one study argues that acute symptomatic seizure is not a rare complication of post COVID‐19 infection. The study observed both new onset seizures and seizures secondary to primary brain insult (e.g., post COVID‐19 encephalitis) in 19 of its 439 patients (4.3%).^68^

Neuropsychiatric outcomes in COVID‐19 survivors were investigated in one study.^69^ One sequela they examined in association with COVID‐19 was Parkinson's disease and parkinsonism. Researchers found some support for an increased incidence of parkinsonism when comparing the whole COVID‐19 cohort (0.11%) to those with more severe COVID‐19 in the intensive therapy unit (0.26%). However, not all hazard ratios were significant, thus more data or further follow‐up are needed to confirm these findings.^69^

Recent studies have explored the cognitive effects of COVID‐19, particularly symptoms resembling ADHD in the adult population. Some patients who recovered from COVID‐19‐pneumonia exhibited cognitive dysfunctions and dysexecutive dysfunction, with difficulties in planning, attention, problem‐solving, and inhibiting inappropriate responses, and issues with verbal memory necessitating further neuropsychological assessment.^70^, ^71^, ^72^, ^73^

## DISCUSSION

This scoping review aimed to evaluate and synthesize the current literature on neuropsychiatric manifestations in patients diagnosed with COVID‐19. The most frequently reported post‐COVID‐19 neuropsychiatric disorders were neurocognitive deficits, anxiety, and depression, which were often co‐diagnosed. PTSD and insomnia, along with related sleep disturbances, were also commonly reported. Our review highlights the importance of considering less commonly reported neuropsychiatric manifestations, such as eating disorders and schizophrenia. The review underscores the necessity for comprehensive neuropsychiatric assessment in post‐COVID‐19 patient management, with particular attention to both common and less frequently observed conditions.

The findings in this paper align closely with existing literature. One study reported persistent cognitive dysfunction following COVID‐19 infection, with some individuals experiencing symptoms for up to a year. In that study, verbal memory was the most significantly affected cognitive domain, and approximately 30% of patients were diagnosed with PTSD and depression.^74^ Similarly, our research identified a high prevalence of neurocognitive deficits, depression, and trauma‐related disorders. While the aforementioned study primarily focused on the longitudinal prevalence and trajectory of cognitive deficits, our study concentrated on identifying the most prevalent deficits over time and exploring the potential for other neuropsychiatric issues that may arise in patients post COVID‐19.

A narrative review examined neuropsychiatric complications of long COVID up to March 2022, revealing similar clinical manifestations and complications compared to our scoping review, although differences were noted in the frequency of the most common symptoms. The narrative review identified fatigue as the most prevalent neuropsychiatric manifestation, whereas our study found neurocognitive deficits, such as memory loss, to be the most common complications.^75^ Both studies also reported significant symptoms of insomnia, sleep‐related disorders, anxiety, depression, and PTSD, in addition to fatigue and neurocognitive deficits.

Furthermore, this review underscores the complexity of how these symptoms manifest in patients and emphasizes the need for further research to identify and manage long COVID complications. Additionally, pre‐pandemic factors like lack of vaccination and lower material wealth were associated with a higher burden of neuropsychiatric symptoms, highlighting the influence of socioeconomic factors on symptom severity.^76^

The duration and prognosis of the diverse symptomatology following COVID‐19 infection is not fully understood, and ongoing research continues to uncover new findings. In this scoping review, most studies reported the presence of neuropsychiatric sequalae following COVID‐19 infection up to years after initial infection. The high prevalence of various neurocognitive deficits as well as depression and anxiety underscore the profound impact that COVID‐19 has on public mental health.

As more is learned about COVID‐19 infection and its neuropsychiatric sequelae, clinical guidelines must adapt quickly to ensure timely diagnosis and management. In the interim, early detection of these sequelae is paramount. This highlights the importance of multidisciplinary teams in management. Psychiatric and psychological care will play a significant role in the multidisciplinary approach, and healthcare systems may consider expanding capacity and access through resource allocation and telemedicine services.

The strength of this review is bolstered by the number of studies and variety of neuropsychiatric sequelae included. In 333 studies, over 20 different post‐COVID‐19 neuropsychiatric diagnoses were systematically reviewed, with the most prevalent being neurocognitive deficits, anxiety, and depression, and PTSD. While comprehensive in quantity and diagnostic variety, this scoping review was limited to somatic symptoms and mental health sequelae of COVID‐19. The review did not explore other dimensions like the impact of social determinants and its interactions with mental health. Moreover, the inclusion criteria for the studies were limited to English‐language publications only. Furthermore, the reliability of results may be influenced by measurement bias in studies that utilized self‐reported questionnaires or gathered follow‐up data through telephone interviews.

## CONCLUSIONS AND RECOMMENDATIONS

This comprehensive review of the literature highlights the significant neuropsychiatric and psychiatric sequelae associated with COVID‐19. Patients diagnosed with COVID‐19 exhibit a markedly elevated risk of developing various mental health disorders, including schizophrenia spectrum and other psychotic disorders, depressive disorders, anxiety and obsessive‐compulsive disorders, PTSD, sleep‐wake disorders, and cognitive impairments. These risks persist for extended periods, with some symptoms remaining prevalent up to 2 years post diagnosis. The severity of the initial infection, especially in ICU‐admitted patients, appears to correlate strongly with the likelihood and severity of subsequent mental health issues. Neuroimaging studies corroborate these findings, revealing structural and functional brain changes associated with depressive symptoms. Additionally, the presence of biomarkers such as inflammatory markers and cytokines further elucidates the pathophysiological mechanisms underlying these neuropsychiatric conditions. To support COVID‐19 survivors effectively, healthcare systems should implement systematic long‐term follow‐up and monitoring programs, establish multidisciplinary teams to address varied mental health needs, and develop targeted screening protocols for high‐risk groups to facilitate early detection and intervention.

Moreover, promoting further research on the long‐term neuropsychiatric effects of COVID‐19 and the effectiveness of therapeutic approaches is crucial. Public health policies should prioritize mental health support, integrating services into post‐COVID‐19 care plans and allocating resources to bolster mental health infrastructure. Increasing awareness among healthcare providers and educating patients and families about potential long‐term neuropsychiatric sequelae will ensure comprehensive mental health assessments and prompt intervention when symptoms arise.

## AUTHOR CONTRIBUTIONS

**Wali Yousufzai:** Conceptualization; development of methodology; formal analysis; resources; writing—original draft preparation; writing—review and editing; visualization; project administration. **Alex Heo:** Writing—original draft preparation; visualization. **Kyle Gu:** Writing—original draft preparation. **Edward Sun:** Writing—original draft preparation. **Gabriel Lopez:** Writing—original draft preparation. **Shreya Balamurali:** Writing—original draft preparation. **Jennifer Adjei‐Mosi:** Writing—original draft preparation. **Riley Shin:** Writing—original draft preparation. **Daniel B. Stuart:** Development of methodology; formal analysis; resources. **Peggy Edwards:** Development of methodology. **Regina Baronia:** Resources; writing—review and editing. **Wail Amor:** Supervision. **Terry McMahon:** Writing—review and editing; supervision. All authors have read and agreed to the published version of the manuscript.

## CONFLICT OF INTEREST STATEMENT

The authors declare no conflicts of interest.

## ETHICS APPROVAL STATEMENT

N/A.

## PATIENT CONSENT STATEMENT

N/A.

## CLINICAL TRIAL REGISTRATION

N/A.

## Data Availability

Study protocol is available in Open Science Framework at osf.io under the project name “A Scoping Review‐First Episode of Psychiatric and Neuropsychiatric Disease Among Patients Infected with Covid‐19”, registration https://doi.org/10.17605/OSF.IO/5VJN2.
